# Cancers and the War

**Published:** 1918-12

**Authors:** 


**Cancers and the War**. By M. Em. Forgue. *Bulletin de l'Académie de Médecine.* July 16, 1918.

The author discusses the two following questions :

1) Which are the circumstances of the war that may theoretically be considered as liable to develop cancers among the soldiers?

2) How frequent did cancers prove in the army during the war?

1) In many cases, trauma seems responsible for the development of tumors. However an acute and *single* trauma seems unable to develop a malignant tumor in a healthy organism with no predisposition; generally the trauma aggravates some preexisting tumor. *A single* trauma might only be responsible, as it seems, for the development of sarcoma of the bones; tumors of the epithelioma variety seem to develop only after repeated, chronic, irritating causes.

Among these local causes to be found in war circumstances are:

*a*) The chronic cutaneous irritation due to want of cleanliness of the skin, and exposure to bad weather.

*b*) The chronic inflammations of some ancient scars or fistula.

*c*) The irritating effects on mouth and tongue due to tobacco, neglected teeth, the use of spirits, syphilis....

2) To have some idea as to the frequency of cancers among the soldiers, the author examined the notes of the medical committee directed to grant annuities to the disabled soldiers. It is astonishing how few demands emanate from soldiers suffering from cancers. On the whole, there were only 486 of these, among 200,000 and more, until April 1918. Half of these cases concerned cancers of the stomach. Next in frequency came cancers of the liver, and then cancers of the bowels, the esophagus, and the rectum. Cancers of the tongue proved also rather frequent. Until the age of 30, cancers seem rare among the soldiers; they develop especially between 40 and 45.

As to the medico-legal point of view, the committees generally admit of some connection between the cancers and the war circumstances.

